# Tailoring Drug Release Properties by Gradual Changes in the Particle Engineering of Polysaccharide Chitosan Based Powders

**DOI:** 10.3390/polym9070253

**Published:** 2017-06-29

**Authors:** Ednaldo G. do Nascimento, Lilia B. de Caland, Arthur S.A. de Medeiros, Matheus F. Fernandes-Pedrosa, José L. Soares-Sobrinho, Kátia S.C.R. dos Santos, Arnóbio Antonio da Silva-Júnior

**Affiliations:** 1 Laboratory of Pharmaceutical Technology and Biotechnology, Department of Pharmacy, Federal University of Rio Grande do Norte, UFRN, Gal. Gustavo Cordeiro de Farias, Petropolis, Natal 59072-570, RN, Brazil; ednaldogn40@gmail.com (E.G.d.N.); liliabasiliocaland@gmail.com (L.B.d.C.); arthursergiomedeiros@gmail.com (A.S.A.d.M.); mpedrosa@ufrnet.br (M.F.F.-P.); 2 Department of Pharmacy, Center of Health Sciences, Federal University of Pernambuco, Professor Moraes Rego 1235, Recife 50670-901, PE, Brazil; joselamartine@hotmail.com; 3 School of Pharmaceutical Sciences, Federal University of Amazonas, UFAM, General Rodrigo Octávio Jordão Ramos, 6200, South Sector, Manaus 69077-000, AM, Brazil; katiasolange@hotmail.com

**Keywords:** polysaccharides, chitosan, microparticles, spray drying, structural properties, polymer characterization, drug release

## Abstract

Chitosan is a natural copolymer generally available in pharmaceutical and food powders associated with drugs, vitamins, and nutraceuticals. This study focused on monitoring the effect of the morphology and structural features of the chitosan particles for controlling the release profile of the active pharmaceutical ingredient (API) propranolol hydrochloride. Chitosan with distinct molecular mass (low and medium) were used in the formulations as crystalline and irregular particles from commercial raw material, or as spherical, uniform, and amorphous spray-dried particles. The API–copolymer interactions were assessed when adding the drug before (drug-loaded particles) or after the spray drying (only mixed with blank particles). The formulations were further compared with physical mixtures of the API with chitin and microcrystalline cellulose. The scanning electron microscopy (SEM) images, surface area, particle size measurements, X-ray diffraction (XRD) analysis and drug loading have supported the drug release behavior. The statistical analysis of experimental data demonstrated that it was possible to control the drug release behavior (immediate or slow drug release) from chitosan powders using different types of particles.

## 1. Introduction

**Figure 1:**
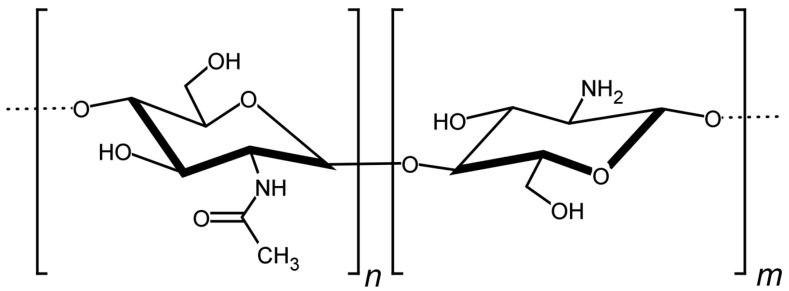
Schematic presentation of chemical structure of the copolymer chitosan composed of *N*-acetylglucosamine (2-acetamido-2-deoxy-d-glucose) [n] and glucosamine (β (1–4)-linked 2-amino-2-d-glucose) [m].

Polysaccharides have been extensively used as ingredients in the pharmaceutical and food industries. The chitosan is a cationic polysaccharide copolymer composed of glucosamine (β (1–4)-linked 2-amino-2-d-glucose) and *N*-acetylglucosamine (2-acetamido-2-deoxy-d-glucose) (Figure 1). This raw material is generally produced by the partial deacetylation of chitin, the second most abundant polymer in nature, present in exoskeletons of crustaceans [1,2]. In solid dosage forms, the chitosan has been used as excipient for tablets [3,4], diluent or granulating agent [5,6]. In liquid dispersions, this copolymer is also used as an emulsifying or thickness agent [7,8]. Furthermore, it has been applied in drug delivery systems for modulating the release or enhancing the solubility of pharmaceutical ingredients [9,10,11].

Chitosan based microparticles have also been used for drug targeting in specific tissues such as gastric or colon mucosa [12,13]. This copolymer is responsible for enhancement of drug permeability and bioavailability, due to the mucoadhesive properties [14]. Added to their versatile properties, chitosan is a biomaterial biodegradable with safety established for the oral rout in humans [15,16,17]. The chitosan particles can be produced using several methods, such as freeze-drying [18], spray-drying [19], ionotropic gelation, coacervation or co-precipitation with solvent evaporation [18,20,21]. The desired properties of particles such as size, shape, density, porosity and the drug-loading ability are certainly considered in the experimental design [22]. The spray drying is a single step method, widely used in foods, able to produce small, uniform and spherical polymeric particles with the drug generally homogeneously dispersed into the polymeric matrix [19,23].

Considering the active pharmaceutical ingredient (API) release rate for oral rout, the pharmaceutical dosage forms are classified as the immediate release, in which generally 80% *w*/*w* of the ingredient is released until 45 min in acid medium. The modified drug delivery devices can present the drug release as being pH-tunable or slow drug release in a superior interval time. Taking into account the hydrogel behavior of chitosan particles, the drug release rate can be modulated by the pH of medium or by inherent copolymer properties such as degrees of deacetylation (DD) and molecular mass [24,25]. Other aspects involve the structural properties of particles, drug crystallinity, and drug–copolymer interactions.

In this study, chitosan with different viscosimetric molecular mass (low and medium) were used to evaluate how the morphology of particles (commercial raw material or spray-dried particles) and drug–copolymer interaction in physical mixtures or in drug-loaded spray-dried microparticles affected the drug release rate. For this purpose, the anti-hypertensive propranolol hydrochloride was used as a pharmaceutical ingredient due to high water solubility. The physicochemical and structural features of different powder samples were carefully monitored and the drug dissolution data subjected to comparisons using a suitable mathematical modeling approach. 

## 2. Materials and Methods 

### 2.1. Materials

Low and medium molecular mass chitosan (LMC and MMC, respectively) were purchased from Sigma-Aldrich (São Paulo, SP, Brazil). Propranolol hydrochloride (PPHy) and microcrystalline cellulose (MCC) were from SM pharmaceutical enterprises (São Paulo, SP, Brazil). Chitin (CH) was from Polymar (Fortaleza, CE, Brazil). All other reagents were of analytical grade. The purified water (1.2 μS) was prepared from an OS50 LX reverse osmosis purification apparatus Gehaka (São Paulo, SP, Brazil).

### 2.2. Physicochemical Characterization of Chitosan

The viscosimetric molecular mass (Mv) of chitosan was determined using the flow time of six chitosan solutions at different concentrations (0.17; 0.18; 0.19; 0.21 and 0.23% *w*/*v*) diluted in acetic acid 0.5 M/sodium acetate 0.2 M buffer. The dispersions passed through capillary viscosimeter CFRC-100 model (Cannon-Fenske Routine, USA), with size 100 at 25 ± 0.1 °C (*n* = 6). The relative (ηr), specific (ηsp), reduced (ηred) and inherent (ηinh) viscosities were determined, as shown below in Equations (1)–(4).

ηr = t/t0
(1)

ηsp = ηr − 1
(2)

ηred = ηsp/C
(3)

ηinh = ln(ηr)/C
(4)

where t and t0 represent flow time of chitosan solutions and buffer solutions, respectively, C represents the natural logarithmic chitosan concentration.

The ηred and ηinh values were plotted versus chitosan concentration and the intrinsic viscosity (η) were determined by the curve extrapolation for the absence of chitosan. Therefore, the correlation of molecular mass with the η value were determined by the empirical equation of Mark-Houwink-Sakurada Equation (5), where [η] is the intrinsic viscosity, *k* and *a* are constants for a specific polymer solution with the used solvent at a specific temperature, that is *k* = 3.5 × 10^−4^ and *a* = 0.76 [26].

[η] = *k*Mv*a* (5)

The degree of deacetylation was determined using the conductometric titration method [27]. The protonated amine groups were determined in chitosan solution of 0.5% *w*/*v* dissolved with aqueous hydrochloric acid of 0.06 M, adding in the following NaOH solution (0.15 M) to allow the deprotonation of chitosan. The degree of deacetylation was measured according the following Equation (6):

DD = 100 MA (ΔV . C_NaOH_/ΔV . C_NaOH_ . ΔM + WCHIT . ms) (6)

where DD is the degree of deacetylation, MA is the molecular mass of acetylated copolymer, ΔV is the variation of NaOH volume, ΔM is the difference between the molecular mass between acetylated and deacetylated copolymers, WCHIT is the solid mass fraction of chitosan and ms is the mass of the sample.

### 2.3. Preparation of Powder Samples

#### 2.3.1. Spray-Dried Microparticles

The aqueous solution (acetic acid 1.0% *w*/*v*) containing chitosan at 0.5% (*w*/*v*) was prepared under magnetic stirring for 24 h and dried in an ADL311S spray dryer (Yamato Scientific Co., Tokyo, Japan) to obtain the blank microparticles. For the drug-loaded chitosan microparticles, the propranolol was dissolved with the copolymer at mass ratio drug-chitosan of 1:2. The drying conditions were previously established in previous studies [28]. The inlet temperature of 140 °C, outlet temperature of about 90 °C, air pressure of 0.1 MPa, air flow of 0.32 m^3^/min and a feed flow rate of 5 mL/min through a nozzle of 0.4 mm were used during the experiments.

#### 2.3.2. Powder Formulations 

The hard gelatin capsules n° 2 were filled with powder mixtures containing propranolol hydrochloride. The drug was physically mixed with the different excipients using mortar, or loaded in the spray-dried chitosan microparticles. The final composition of different samples are shown in Table 1.

### 2.4. Morphology, Particle Size and Surface Area Analysis

The shape and surface aspect of particles were accessed using the scanning electron microscopy (SEM) images taken in TM 3000 Microscope Hitachi (Tokyo, Japan). The particles were dried and mounted on metal stubs using double-sided adhesive carbon tape and analyzed at the voltage of 20.0 kV. The mean diameter and the size distribution of the microparticles was determined using dynamic light scattering (DLS) in a Nanotrac NPA252 (Montgomeryville, PA, USA) with Flex software 10.4.3. The amount of 4.0 mg of powder was dispersed in 15.0 mL of aqueous solution of polysorbate 80 at 0.5% *w*/*v*. The cumulative diameter of 10, 50 and 90% in the particle size distribution were determined in triplicate. The index span was calculated by the equation: SPAN = D90 − D10/D50 [28].

The surface area, pore volume and pore size of the chitosan microparticles were determined following the method of Brunauer-Emmett-Teller (BET), using the liquid N2 adsorption and desorption isotherms, measured at 77 K temperature with an ASAP 2420 surface area analyzer (Micromeritics Instrument, Norcross, USA). All samples were degassed and stored at vacuum at room temperature overnight prior to measurements. The experiments were repeated at least three times using fresh powder.

### 2.5. X-ray Diffraction Analysis

The X-ray diffraction (XRD) analysis was performed for pure compounds, physical mixtures and spray-dried microparticles in a D2 Phaser diffractometer (Bruker corporation, Billerica, USA) using CuK radiation (λ = 1.54 Å) with a Ni filter. The measurements were performed in a 2-Theta angle variation of 5–45°, angular step of 0.004° and 30 kV voltage.

### 2.6. Fourier Transform Infrared Spectrophotometry (FT-IR) Studies

The FT-IR was recorded in an IR Prestige-21 equipment (Shimadzu, Kyoto, Japan). Samples of 2 mg of powder were mixed with 300 mg of potassium bromide (KBr) for pellets confection, using a hydraulic press at 10 KgF. The middle infrared region (MID) was analyzed in the range of 400–4000 cm^−1^.

### 2.7. Drug Loading Analysis

The spray-dried PPHy-loaded chitosan microparticles were massed to contain 20 mg of drug and then dissolved with 20 mL of purified water. Then, the methanol was added to a final volume of 100 mL with a volumetric flask. The measurements were done in triplicate by UV/Vis spectrophotometry at a wavelength of 290 nm. The absorbance results were correlated with drug concentration through a previously determined calibration curve. The drug loading efficiency was calculated from the relationship between the analytical and the theoretical drug contents.

### 2.8. Drug Dissolution Assays 

The dissolution profile of samples was performed in the simulated gastrointestinal medium [29], using a dissolution equipment 299 model (Ethiktechnology, Sao Paulo, Brazil). The hard gelatin capsules containing the formulations were placed in basket apparatus, using 1000 mL of hydrochloric acid solution 1% (*v*/*v*) as dissolution medium at 37 ± 0.5 °C, under 100 rpm agitation. At specific intervals, aliquots of 10 mL of dissolution medium were removed, filtered through a 0.45 µm cellulose acetate, and analyzed by UV spectrophotometry at 290 nm, previously validated in a spectrophotometer 60S Evolution (Thermo Fisher Scientific Inc, Madison, WI, USA). All experiments were performed six times (*n* = 6) and the cumulative percentage of released propranolol was plotted versus time. 

The effect of the experimental variations on the drug release profile of the different samples was evaluated by comparisons using the independent-model of similarity factor (f2), according to Equation (7).

F2 = 50·log [1 + (1/n)·∑ (D1t − D2t) 2] −0.5]·100
(7)

where, n is the number of experimental intervals, t is the interval time, D1t is the drug ratio dissolved at a specific interval time for formulation (1), and D2t is the drug ratio dissolved at specific interval time for formulation (2). Formulations with drug dissolution profiles that are statistically similar present F2 values in the range of 50 to 100.

### 2.9. Statistical Analysis

The analytical data were expressed as the mean ± standard deviation. The Mann Whitney test was applied for pairwise comparisons, while multiple comparisons were assessed by the analysis of variance (ANOVA) on ranks (Kruskal Wallis test) at 0.05 of significance level.

## 3. Results

### 3.1. Physicochemical Characterization of Chitosan

**Figure 2:**
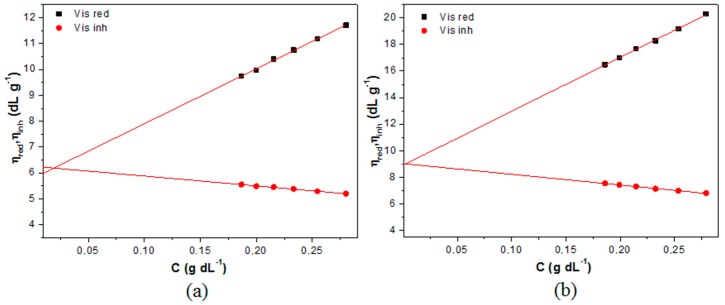
The reduced (ηred) and inherent viscosities (ηinh) as function of chitosan concentration (C) for the two used copolymers: (**a**) low molecular mass chitosan (LMC) and (**b**) medium molecular mass chitosan (MMC).

The different used chitosan (LMC and MMC) have the viscosimetric molecular mass (Mv) and degree of deacetylation assessed to control the behavior of copolymers. The intrinsic viscosity [η] was determined extrapolating the intersection in the plot of the reduced (ηred) and inherent viscosities (ηinh) as a function of the chitosan concentration (Figure 2). The calculated Mv values for LMC and MMC were of 4.61 × 10^5^ and 7.15 × 10^5^ g·moL^−1^, respectively, which were estimated using the Mark-Houwink-Sakurada equation (Equation (1)).

**Figure 3:**
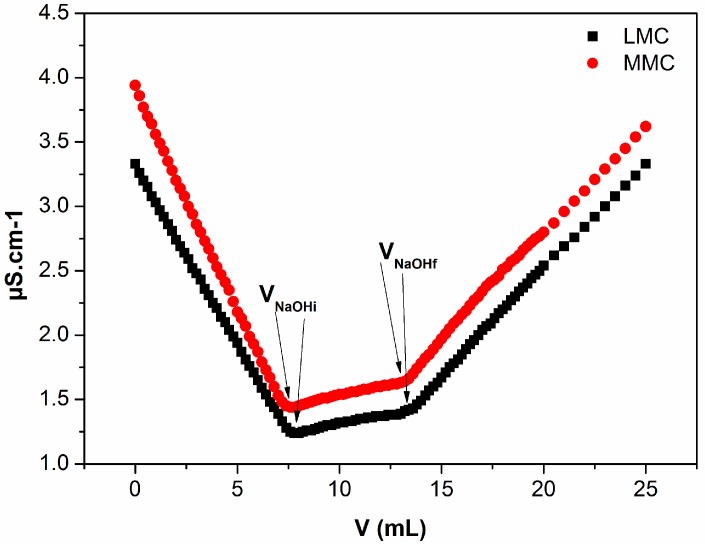
Conductivity titration curves as a function of added volume of NaOH solution (0.15 M) for each used chitosan.

Figure 3 shows the conductivity curves for the MMC and LMC. The first linear descending branch of the curve is the neutralization of excess acid used to solubilize the copolymer. The second was due to the neutralization of NH_3_^+^ of chitosan amino groups. Finally, the ascending points represent the excess alkaline solution (OH^−^) after the equivalence point. The extrapolation of these three lines made it possible to observe two inflection points, corresponding respectively to the volume of initial and final NaOH volume (VNaOHi and VNaOHf) required to neutralize the protonated amino group of chitosan [30]. The degrees of deacetylation of 88.16% (±0.18%) and 88.74% (±2.63%) were determined for the LMC and MMC, respectively.

### 3.2. Physicochemical Properties of Particles

The microparticles were successfully produced using the selected spray drying parameters. They were fine and dried powders, with yield levels of about 56.49 ± 0.06% for the blank microparticles and of about 57.29 ± 0.09% for drug-loaded microparticles. The encapsulation efficiency ranged between 90.1 ± 0.7 and 91.0 ± 3.0 for the PPHy-LMC-MPs and PPHy-MMC-MPs, respectively. Table 2 shows the data of particle size and BET analysis for the spray dried microparticles. The mean diameter ranged between 1.72 to 3.45 μm (SPAN index between 1 and 3).

**Figure 4:**
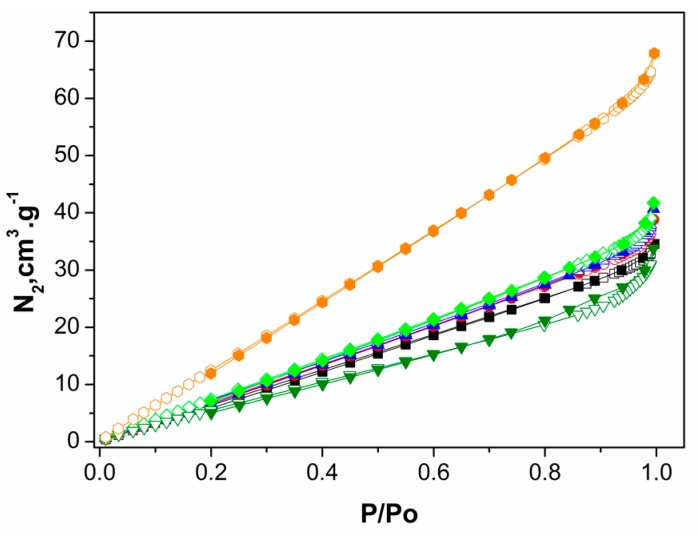
The N_2_ adsorption-desorption isotherms for different chitosan powder samples LMC (■); MMC (●); blank LMC-MPs (▲); blank MMC-MPs (♦); PPHy-LMC-MPs (▼); PPHy-MMC-MPs (●).

Table 2 also shows the BET parameters calculated from the nitrogen adsorption–desorption isotherms for different samples of chitosan powders (Figure 4). No hysteresis was observed and the adsorption increased linearly at relative pressure in the range of 0.0–1.0. The drug loading seems to have slightly decreased the pore size for the two tested copolymers.

**Figure 5:**
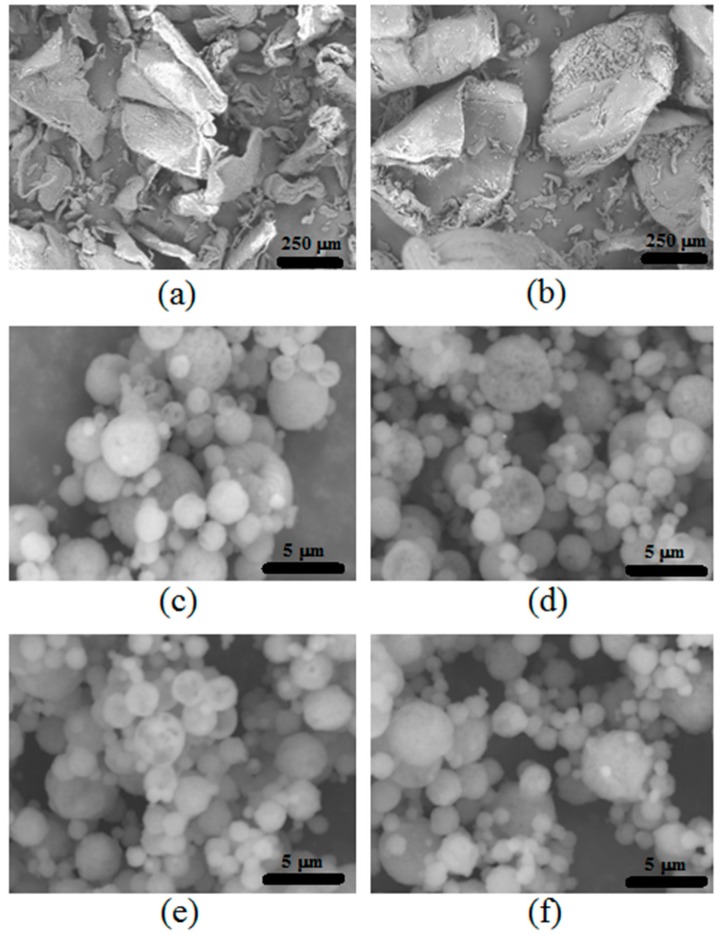
Scanning electron microscopy (SEM) images of different chitosan particles: (**a**) LMC, (**b**) MMC, (**c**) Blank LMC-MPs, (**d**) Blank MMC-MPs, (**e**) PPHy-LMC-MPs, and (**f**) PPHy-MMC-MPs.

**Figure 6:**
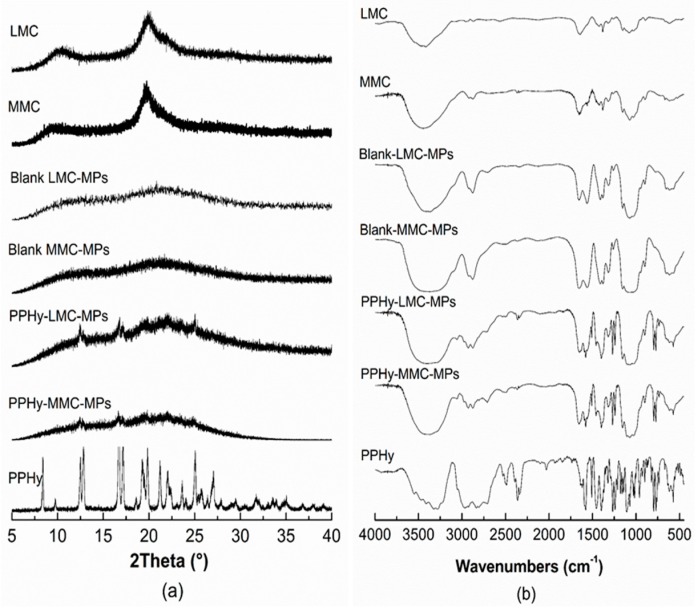
(**a**) X-ray diffraction (XRD) patterns and (**b**) Fourier transform infrared (FT-IR) spectra for different chitosan powders.

Figure 5 shows the SEM images for the distinct chitosan particles. The irregular shape with wide particle-size distribution for the two commercial chitosan raw materials LMC and MMC are shown in Figure 5a,b, respectively. In contrast, the blank (Figure 5c,d) and drug-loaded spray-dried microparticles (Figure 5e,f) show regular spherical particles with narrow particle size distribution, a signature of spray dried particles [31]. This fact is important for the release rate that the hydrogel powder can supply, consequently affecting the biological activity. Another structural feature that controls the drug release from the particles is the crystallinity of the compounds. This aspect was assessed using XRD analysis (Figure 6a).

The FT–IR spectra (Figure 6b) of the commercial chitosan raw materials showed that the characteristic absorption bands of O–H and N–H stretch appeared overlapped in a large and broad band at 3450 cm^−1^. The C–H stretch of methyl groups at 2890 cm^−1^, C=O amide stretch at 1655 cm^−1^, the N–H angular deformation bands at 1583–1594 cm^−1^, the C–H symmetric angular deformation bands of methyl at 1380–1383 cm^−1^, the C–N amino stretch at 1308–1380 cm^−1^ and finally the C–O secondary alcohol stretch at 1103 cm^−1^. The propranolol spectra showed O–H stretch at 3283 cm^−1^, the C=C aromatic stretching at 1705 cm^−1^, the N–H angular deformation at 1590 cm^−1^, the C–O ether stretch at 1270–1240 cm^−1^ and the C–O secondary alcohol stretch at 1103 cm^−1^. 

The FT–IR of drug-loaded particles did not show any relevant shift of recorded stretches for the pure compounds. The bands of drug and copolymer appeared overlapped, suggesting that the drying process did not induce any chemical reaction of the drug with chitosan. 

### 3.3. Drug Dissolution Assays 

**Figure 7:**
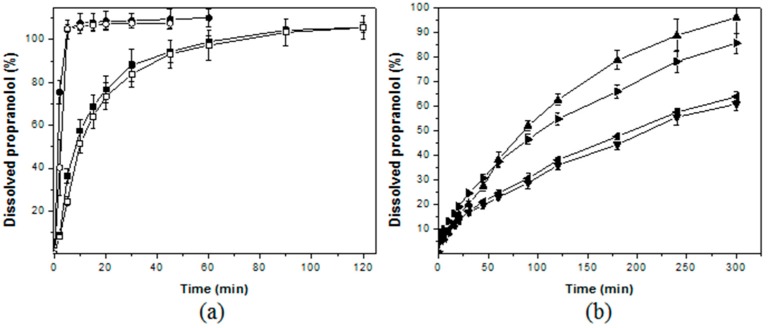
Dissolution profile of propranolol from different chitosan powder formulations: (**a**) LMC (■), MMC (□), Chitin (●), and microcrystalline cellulose (○), and (**b**) blank LMC-MPs (▲), blank MMC-MPs (►), PPHy-LMC-MPs (▼), and PPHy-MMC-MPs (◄).

Before the dissolution assay, all produced formulations of the capsule have the weight variation and drug content analytically controlled. According to the quality control of pharmaceutical and food products, a variation within the ±10% for average weight and drug content, with relative standard deviations of less than 4%, were given as acceptable. Figure 7 shows the drug dissolution profile from different formulations of powders. Figure 7a plots the formulations that exhibited the immediate drug release profile, which released more than 80% before 40 min [29]. The drug release profile from powders containing the two distinct commercial chitosan microparticles were further compared with powder mixtures containing chitin or microcrystalline cellulose due to their use as diluent for solid dosage forms. The mixtures with microcrystalline cellulose (MCC) and chitin showed similar behavior, releasing the full drug content before 10 min. The mixtures with chitosan raw materials as diluents with distinct molecular mass (LMC and MMC) exhibited a slight delay, explained due to the hydrogel behavior of this compound compared with the MCC and chitin that are water insoluble excipients. After 30 min, the capsules containing LMC and MMC dissolved the drug content of 87.0 ± 1.0% and 87.1 ± 2.7%, respectively.

Figure 7b shows the powder formulations with slow drug release profiles. The simple mixture of drug with distinct blank microparticles was able to modulate the drug release, enhancing about 3 times the necessary time to release 100% of drug in the dissolution medium. The drug release rate was further slowed for the drug-loaded spray-dried chitosan microparticles. These samples release about 50% of drug after 300 min. The formulations prepared with the distinct chitosan were also compared. The different molecular mass (LMC and MMC) did not induce relevant changes in the drug dissolution profiles. The comparisons of the experimental data using the similarly factored treatment (f2 > 50) showed the statistical similarity for the pairs LMC with MMC, blank LMC-MPs with MMC-MPs, and PPHy-LMC-MPs with PPHy-MMC-MPs, observed in Table 3.

## 4. Discussion

In this study, we have used two copolymers with the same deacetylation degree, but with medium and low molecular mass, that governed important physicochemical and functional properties of chitosan particles. These aspects of the chitosan were successfully characterized before to explore the physical aspects of particles (Figure 2 and Figure 3). Moreover, we considered morphological and physical aspects of particles, e.g., the irregular commercial chitosan, or spherical spray-dried chitosan microparticles. The drug–copolymer interactions were also assessed in the simple mixtures or from the drug-loaded spray-dried microparticles, as a strategy to modulate the drug release properties for application in foods, cosmetics, chemicals and pharmaceuticals.

The data in Table 1 demonstrated the small and narrow-sized spray dried microparticles compared with previous studies [32,33,34]. The MMC induced the formation of larger particles compared with LMC, due to the higher packing of smaller polymer chains [35]. In addition, the particle size is highly affected by the viscosity of feed solution, consequently affecting the droplet formation during the drying process. However, these differences did not affect the high drug loading for both samples of spray-dried microparticles. Previous studies also reported spray-dried chitosan microparticles with mean diameters of approximately 3 μm, using low molecular chitosan solutions at 0.5 to 1.0% (*w*/*v*) [36,37].

The experimental data of isotherms (Figure 4) were applied to BET calculations to determine surface area of particles (Table 2). Curves typical of mesopore-rich materials were observed, with pore diameters between 20 and 50 Å [38]. The BET analysis showed that both LMC-MPs and MMC-MPs showed equivalent surface area and pore volume. Regarding the drug-loaded particles, PPHy-LMC-MPs exhibited lower surface area and pore volume than PPHy-MMC-MPs, demonstrating a more efficient packing of the copolymer with lower chains in the particles. The greater surface area of PPHy-MMC-MPs compared with PPHy-LMC-MPs did not affect the high EE% of microparticles, since similar values were approximately 90%. This parameter was previously affected by the surface characteristics of chitosan particles produced using different microencapsulation methods [39]. This fact reinforces the amazing performance of the spray drying to produce chitosan microparticles for slow release of small molecules, especially due to the water penetration and swelling of polymeric matrix.

Figure 5 shows the SEM images for the distinct chitosan particles. The irregular shape with wide particle-size distribution for the two commercial chitosan raw materials LMC and MMC are shown in Figure 5a,b. In contrast, the blank (Figure 5c,d) and drug-loaded spray-dried microparticles (Figure 5e,f) have shown regular spherical particles with narrow particle size distribution, a signature of spray dried particles [31]. This fact is important for the release rate that powders with hydrogel character can supply, consequently affecting the biological activity. Mys et al. obtained the technique of spray-dried microparticles in the spherical form of syndiotactic polystyrene [40,41].

Another structural feature that controls the drug release from the particles is the crystallinity of the compounds into the particles. This aspect was assessed using XRD analysis (Figure 6a).

The two characteristic reflections from crystalline samples of commercial chitosan raw materials were observed at 10° and 20° for the LMC, and at 10° and 19° for the MMC, respectively, in accordance with previous studies [42,43]. The pure drug also presented the characteristic crystalline diffraction pattern [44]. The spray drying produced amorphous chitosan microparticles. Considering the drug-loaded microparticles, the PPHy was homogeneously dispersed in the polymeric matrix in the amorphous state. The random drug distribution in the particles induces an energetic favorable drug dissolution compared with crystalline particles of drug into polymeric devices. In this approach, the drug dissolution in the chitosan loaded-microparticles offered freely drug–chitosan interactions, which were able to control the drug dissolution. This kind of interaction does not occur when the drug is physically mixed with the chitosan particles. Thus, we can observe interesting differences in drug dissolution behavior. The interactions of drug with the chitosan were assessed using FT-IR analysis (Figure 6b).

The amorphization of chitosan in the blank microparticles caused wavenumber shifts and changes in the transmittance intensity for the C=O amide stretch at approximately 1650 cm^−1^ and for the C–O secondary alcohol stretch at 1103 cm^−1^. This fact was also previously demonstrated [45]. The FT-IR of drug-loaded particles did not show any relevant shift of recorded stretches for the pure compounds. The bands of drug and copolymer appeared overlapped, suggesting that the drying process did not induce any chemical reaction of drug with chitosan.

The adjuvant functionality of chitin, microcrystalline cellulose, chitosan and chitosan microparticles was evaluated, using these components as diluents in propranolol capsules. The drug-loaded microparticles was also encapsulated to observe the drug delivery profile. Before dissolution assay, all produced capsule batches were confronted in weight variation and content uniformity tests (data not shown). All capsule formulations presented the average weight within the ±10% range. Furthermore, the relative standard deviations were less than 4%. Regarding uniformity of content, the results showed that propranolol was assayed within the range 90–110%.

The drug release behavior also showed that the morphology and structural features of the particles also modulate the hydrogel behavior of chitosan, which is pH dependent. The chitosan is soluble at an acidic pH, but the different shape, size, and crystallinity of the spray−dried particles (blank LMC and blank MMC) enhanced the hydrogel behavior of chitosan, decreasing the drug dissolution rate from the powders. Other researchers have observed similar behavior using another polymer (hydroxypropyl methyl cellulose phthalate) in the release of API, verifying that it depends directly on the physical property of the particle and the pH of the medium [46]. The amorphous character and great surface area of these particles compared with the commercial raw chitosan changes the solubility and the swelling rate of the copolymer, changing its interaction with the drug in the aqueous medium [47,48]. In the case of drug-loaded spray-dried chitosan microparticles (PPHy-LMC-MPs and PPHy-MMC-MPs), the drug entrapment in the polymeric matrix further enhanced the drug–copolymer interaction, improving the slow release effect of the powder formulations. The similar dissolution between the pairwise formulations also evidence that the neglectable particle size variations, within 1 µm range between the samples, did not influence the dissolution rate. The particle size directly affects the drug dissolution, in which higher surface area or lower particle size leads to a faster dissolution of the entire particle, as shown in the Noyes-Whitney equation [49]. In the case of polymeric particles with hydrogel character, the slow drug dissolution followed by diffusion from the particle is expected. Therefore, the largest differences in the particle size of hydrogel particles can induce significant differences in drug release kinetic behavior [50].

In this approach, different deacetylation degrees were not tested, because the chitosan commercially ranged from 80 to 85%. Thus, drug release performance from the chitosan powders was dependent on its hydrogel behavior. In addition, the experimental data proved that it was possible to control this property, and consequently the drug–copolymer interaction manipulating the morphology and structural features of the chitosan particles.

## 5. Conclusions

In this study, we have demonstrated the versatility of polysaccharides such as chitosan for food, chemistry and pharmaceutical industry as an ingredient capable of modulating the release behavior of additives, vitamins and active pharmaceutical ingredients. The experimental data discussed in this approach demonstrated that it was possible to modulate the active pharmaceutical ingredient release in chitosan powders to induce an immediate or slow release behavior. This behavior was dependent on the hydrogel behavior of the copolymer, which we have controlled using different types of unprocessed chitosan or spray-dried particles. The mixture of API in the particles also affected the release rate.

## Figures and Tables

**Table 1:** Composition of powders used in the hard gelatin capsules.

Samples	Powder (Composition)	Mass of Diluent (mg)
LMC	Low molecular mass chitosan	76
MMC	Medium molecular mass chitosan	69
CH	Chitin	76
MCC	Microcystalline celullose	76
Blank LMC-MPs	Microparticles of LMC	76
Blank MMC-MPs	Microparticles of MMC	76
PPHy LMC-MPs	Drug-loaded LMC-MPs	116 *
PPHy MMC- MPs	Drug-loaded MMC-MPs	116 *

* 116 mg (76 mg of Chitosan + 40 mg of PPHy). All capsules also contain the addition of 21 mg of talc (diluent) and 40 mg of PPHy.

**Table 2:** Particle size and BET analysis for the spray-dried microparticles.

Samples	Particle Size Analysis					BET Analysis		
	D10 (μm)	D50 (μm)	D90 (μm)	Mean (μm)	SPAN	Surface Area (m^2^/g)	Pore Volume (cm^3^/g)	Pore Size (Å)
Blank LMC-MPs	0.7	2.3	5.2	2.3	2.0	25.3	0.056	42.4
Blank MMC-MPs	1.4	3.5	5.8	3.5	1.3	25.8	0.059	39.7
PPHy-LMC-MPs	0.4	1.7	5.0	1.7	2.7	19.2	0.046	48.7
PPHy-MMC-MPs	1.4	3.4	5.8	3.4	1.3	43.5	0.098	37.5

**Table 3:** Statistical analysis of the drug release behavior.

GROUPS	SAMPLES	Molecular Weight	Q_30min_ ± SD (%)	f2 **
Immediate release	LMC	4.61 × 10^5^	86.95	60.07
	MMC	7.15 × 10^5^	87.15	
	CH	ND	107.60	-
	MCC	3.6 × 10^3^ *	107.04	
Slow release	blank LMC-MPs	ND	20.27	50.35
	blank MMC-MPs	ND	24.58	
	PPHy-LMC-MPs	ND	16.95	77.98
	PPHy-MMC-MPs	ND	14.15	

* Handbook of Pharmaceutical Excipients—Fifty edition; ** similarity factor; ND = not determined.
